# A case report of uncommon giant epidermal inclusion cyst found in the thyroid gland

**DOI:** 10.1097/MS9.0000000000001825

**Published:** 2024-02-15

**Authors:** Hana Mousa, Razan Aljassem

**Affiliations:** a Faculty of Medicine, Damascus University; b Department of ENT, Damascus (Al-Moujtahed) Hospital, Damascus, Syria Arab Republic

**Keywords:** epidermoid inclusion cyst, Giant Cyst, hemithyroidectomy, squamous metaplasia, thyroid gland

## Abstract

**Introduction and importance:**

This case report describes a rare occurrence of an epidermal inclusion cyst (EIC) being found in the thyroid gland and highlights the importance of considering uncommon entities like EIC in the differential diagnosis of thyroid lesions.

**Case presentation:**

A 68-year-old male presented with a large, painless swelling in the anterior neck, causing dysphagia and dysphonia. Imaging and cytology confirmed a benign EIC involving the left lobe of the thyroid, which was successfully removed via hemithyroidectomy, resulting in resolution of symptoms.

**Clinical discussion:**

Epidermoid inclusion cysts are rare in the thyroid gland, with only 16 reported cases worldwide. The cyst was diagnosed through ultrasound-guided fine needle aspiration and confirmed by surgical pathology. Treatment involves complete removal of the cyst and its capsule, which was successfully performed in this case under local anaesthesia with sedation due to the patient’s medical history of COPD.

**Conclusion:**

it is important to consider the possibility of EIC when benign squamous cells are detected in a thyroid aspirate without any follicular cells. In such cases, hemithyroidectomy can be a successful management strategy.

## Introduction

Highlights

- Epidermal inclusion cyst in the thyroid gland is an uncommon condition that may result from squamous metaplasia.
- Symptoms of this condition may vary from those of other cases.
- Imaging diagnostic studies reveal the presence of a cyst in thyroid.
- Diagnosis can only be definitively confirmed through pathology.
- Surgical intervention is recommended for the treatment of these cases.

Epidermal inclusion cysts (EIC) of the thyroid, a rare occurrence in clinical practice, are believed to originate from areas of squamous metaplasia^1^. EICs are most commonly found on the trunk, neck, face, scrotum, behind the ears, and in palmoplantar areas^2^. These cysts can develop as a result of epidermal implantation into the dermis, often following trauma, surgery, or blockage of a pore near a body piercing. They have also been observed in individuals with Gardner’s syndrome, particularly on the head and neck^3^. In cases involving the thyroid, epidermoid cysts typically present as painless, solitary nodules located in the anterior part of the neck that move with swallowing and are not associated with lymph nodes. In addition, dysphagia and dyspnoea can happen if the cyst is extensive^4^. The recommended treatment is a complete surgical resection of the cyst along with its capsule. The recurrence of the lesion following total excision has not been reported in cases in the literature^5^. This case highlights an unusual presentation of a harmless thyroid cyst that is consistent with EIC.

‘This case report has been reported in line with the SCARE Criteria’^6^.

### Case presentation

**Figure 1:**
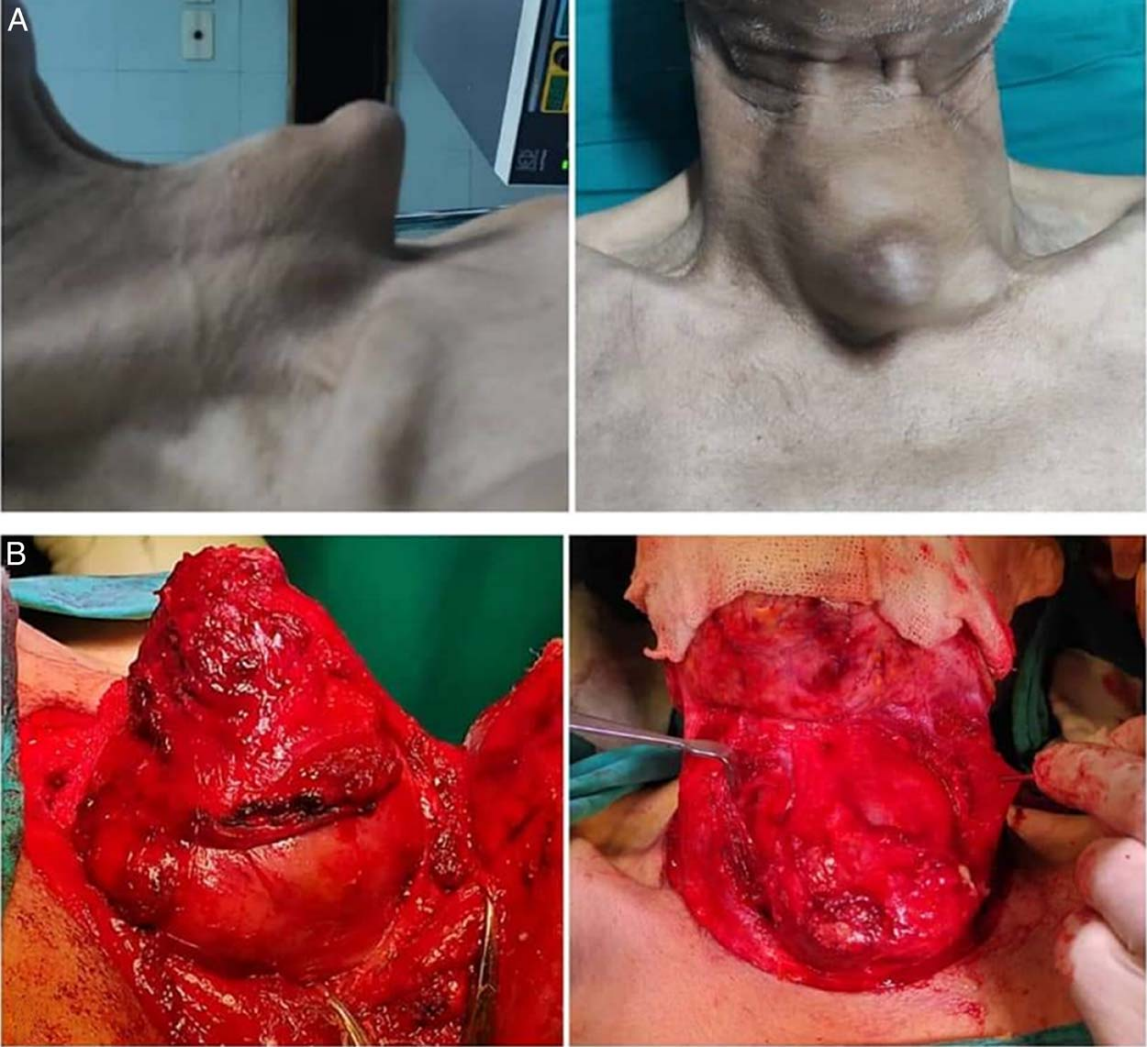
(A) The cyst before the surgery. (B) The cyst during the surgery.

**Figure 2:**
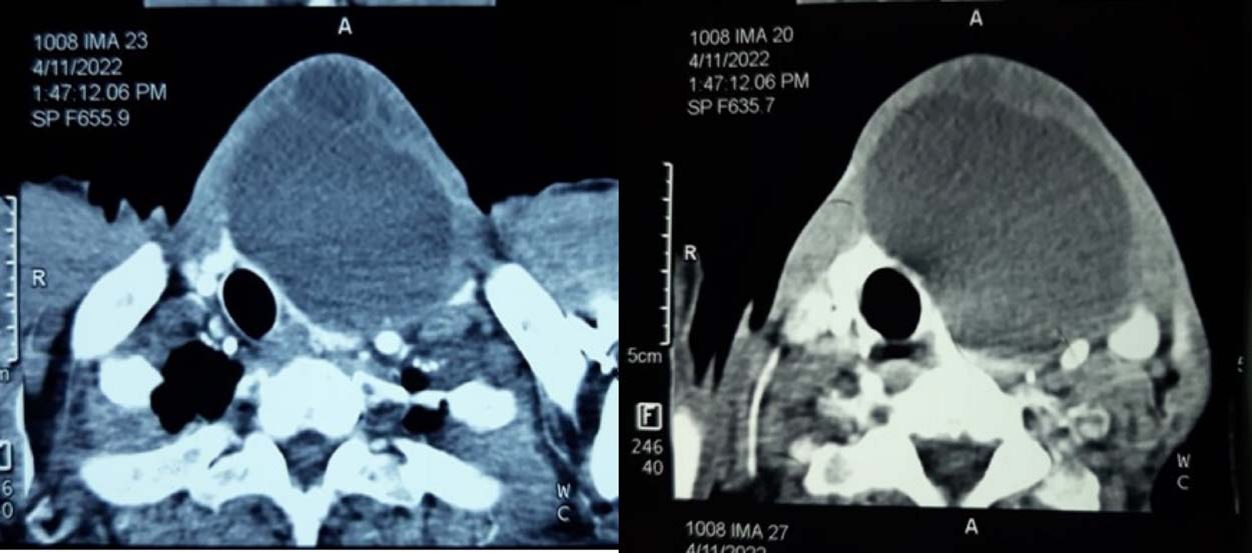
Axial sections revealed a large hypodense cystic lesion involving the left lobe of the thyroid. It pushes the trachea to the right.

**Figure 3:**
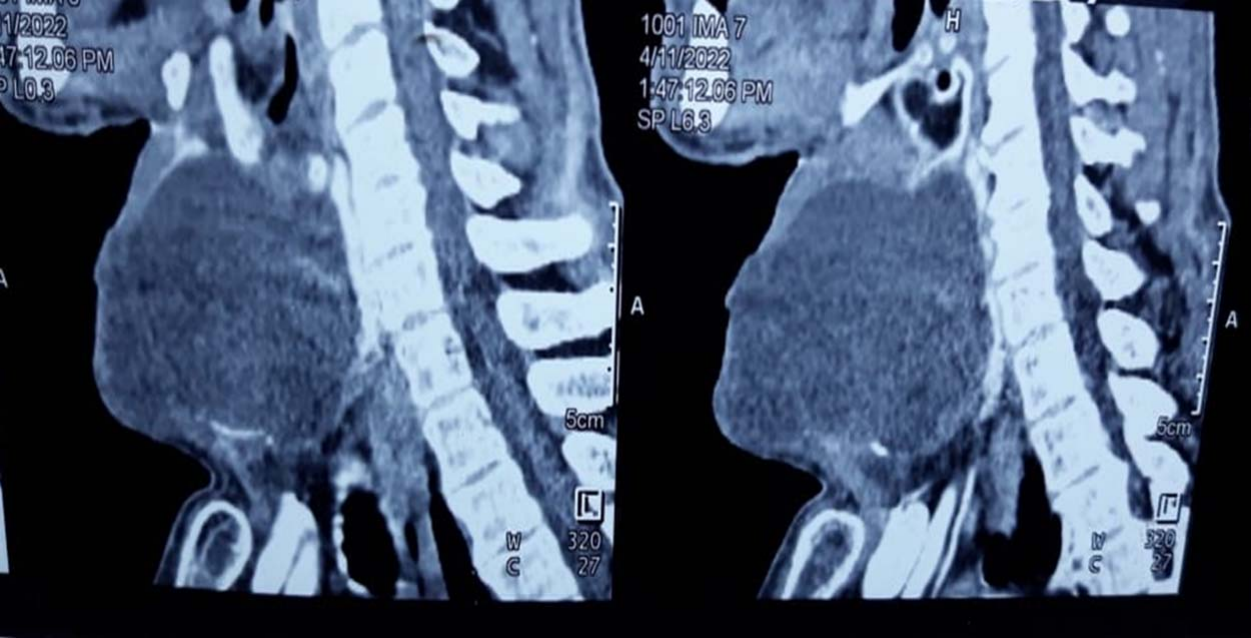
Sagittal sections revealed a large hypodense cystic lesion involving the left lobe of the thyroid.

**Figure 4:**
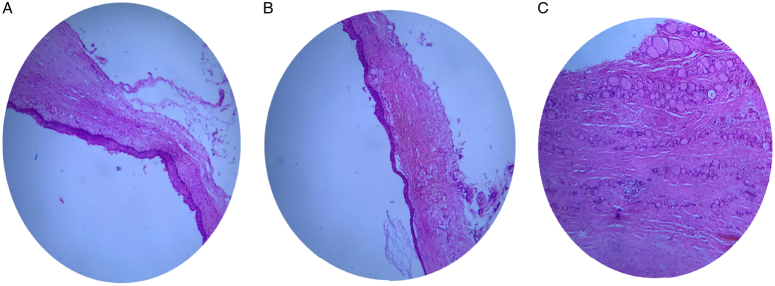
(A) Photomicrograph of the surgical specimen revealed a cyst wall lined by benign squamous epithelium. (B) Rim of compressed benign thyroid parenchyma was observed in the cyst wall, as depicted. (C) This image demonstrated the presence of both normal and atrophic thyroid follicles within the specimen.

A 68-year-old male presented with a 15-year history of a slow-growing swelling in the anterior part of the neck at the midline, along with dysphagia and dysphonia over the last 2 months. He had a medical history of chronic obstructive pulmonary disease, with no reported medications, family history, genetic factors, or psychological issues. On the physical examination, the swelling was small with determined borders, gradually increased in size, and measured 7 × 9 cm (Fig. 1). It was painless, firm in consistency, and moved with deglutination, with no lymphadenopathy. The patient had a clinically and biochemically euthyroid status. Thyroid ultrasound showed a large hypoechoic cyst measuring 7 ×6.7 ×8 cm involving the left side of the anterior neck, compressing and almost replacing the entire left lobe of the thyroid. A contrast-enhanced computed tomography computed tomography (CT) scan of the neck demonstrated a large, hypodense cystic lesion affecting the left lobe of the thyroid along with the isthmus, pressing the trachea to the right (Figs. 2, and 3). Fine needle aspiration cytology (FNAC) was performed multiple times, draining ~30 ml of dirty brown coloured, foul-smelling fluid without a significant reduction in size. Microscopic examination of smears from FNAC indicated degenerated squamous cells and keratinous debris consistent with a benign squamous-lined cyst (EIC) according to the thyroid Bethesda system. The patient underwent hemithyroidectomy, which showed a cyst with solid and cystic areas containing brown cheesy materials measuring 9×6×5 cm. Histopathological examination confirmed a benign thyroid cyst consistent with EIC based on findings of squamous epithelium lining the cyst wall with keratin flakes, covered by fibrous tissue containing normal and atrophic thyroid follicles (Fig. 4). The surgical procedure was performed with local anaesthesia, resulting in the successful removal of the thyroid cyst along with the left thyroid lobe without any complications. Subsequently, the patient was admitted to the ear, nose, and throat (ENT) department for a 2-day postoperative recovery period. Following the removal of the cyst, all compressive symptoms experienced by the patient showed improvement, then referred to an endocrinologist for further evaluation of thyroid condition.

## Discussion

Epidermoid inclusion cysts are rare in the thyroid gland, despite being common throughout the body. Only 16 cases have been reported worldwide^5,7^. The standard sonography appearance of EICs shows them to be unilocular and well-circumscribed. They appear avascular and hypoechoic, similar to subcutaneous fat. FNAC plays a crucial role in the initial diagnosis of near-surface lesions as it helps guide surgical procedures to avoid unnecessary removal of additional tissue^8^. On cytology, they appear as many mature superficial squamous cells with intact nuclei, anucleate squames, clusters of neutrophils and lymphocytes, macrophages, and amorphous debris in the background. It is rare for EICs to undergo malignant transformation, and no such cases have been reported in thyroid cases^9^. This is a picture that is similar to the one obtained in our case. The ages of patients spanned from 4 to 78 years, with females being the most commonly affected. Additionally, the tumours observed varied in size, ranging from 1.0 to 6.0 cm in dimension^7^. In this case, there was a 7 × 9 cm cyst almost completely replacing the left lobe of the thyroid gland, causing compression of the trachea towards the right side. To treat an EIC, it is crucial to remove the cyst along with its capsule completely^2^. In this case, it was decided to remove the thyroid cyst along with the left thyroid lobe under local anaesthesia with sedation. Due to a medical history of chronic obstructive pulmonary disease (COPD), general anaesthesia was not a viable option. Thus, cervical nerve C1, C2, C3, and superior laryngeal nerve blocks are conducted on both sides. The patient was actively involved in decisions about treatment and satisfied with results.

## Conclusion

it is important to consider the possibility of EIC when benign squamous cells are detected in a thyroid aspirate without any follicular cells. In such cases, hemithyroidectomy can be a successful management strategy.

## Ethical approval

Not applicable.

## Consent

Written informed consent was obtained from the patient for publishing this case report and any accompanying images. A copy of the written consent is available for review by the Editor-in-Chief of this journal on request.

## Sources of funding

Not applicable.

## Author contribution

H.M.: design of the study, data collection, data interpretation and analysis, drafting, and critical revision, and the approval of the final manuscript. R.A.: the supervisor, patient care, data collection, and critical revision, and the approval of the final manuscript.

## Conflicts of interest disclosure

Not applicable.

## Research registration unique identifying number (UIN)

Not applicable.

## Guarantor

Razan Aljassem is the guarantor of this work.

## Data availability statement

Not applicable.

## Provenance and peer review

Not commissioned, externally peer-reviewed.
